# Automated Optical Inspection for Defect Identification and Classification in Actual Woven Fabric Production Lines

**DOI:** 10.3390/s22197246

**Published:** 2022-09-24

**Authors:** Chung-Feng Jeffrey Kuo, Wei-Ren Wang, Jagadish Barman

**Affiliations:** Department of Materials Science and Engineering, National Taiwan University of Science and Technology, Taipei 10607, Taiwan

**Keywords:** woven fabric, defect detection, defect classification, support vector machine, image processing

## Abstract

This paper presents a turnkey integrated system that can be operated in real time for real textile manufacturers. Eight types of defects in woven fabric, including stain, broken end, broken weft, hole, nep, double pick, kinky weft and float can be recognized and classified. First, an image is captured by a CMOS industrial camera with a pixel size of 4600 × 600 above the batcher at 20 m/min. After that, the four-stage image processing procedure is applied to detect defects and for classification. Stage 1 is image pre-processing; the filtration of the image noise is carried out by a Gaussian filter. The light source is corrected to reduce the uneven brightness resulting from halo formation. The improved mask dodging algorithm is used to reduce the standard deviation of the corrected original image. Afterwards, the background texture is filtered by an averaging filter, and the mean value is corrected for histogram shifting, so that this system is robust to the texture and color changes of woven fabric. The binary segmentation threshold is determined using the mean value and standard deviation of an image with a normal sample. Stage 2 uses adaptive binarization for separation of the background and defects and to filter the noise. In Stage 3, the morphological processing is used before the defect contour is circled, i.e., four features of each block, including the defect area, the aspect ratio of the defect, the average gray level of the defect and the defect orientation, which are calculated according to the range of contour. The image defect recognition dataset consists of 2246 images. The results show that the detection success rate is 96.44%, and the false alarm rate is 3.21%. In Stage 4, the defect classification is implemented. The support vector machine (SVM) is used for classification, 230 defect images are used as training samples, and 206 are used as test samples. The experimental results show that the overall defect recognition rate is 96.60%, providing that the software and hardware equipment designed in this study can implement defect detection and classification for woven fabric effectively.

## 1. Introduction

Due to the different methods of interweaving, the fabric is divided into two types: woven and knitted. Compared to knitted fabrics, woven fabrics with interlaced warp and weft are more stable and less prone to deformation, making them more widely used in the market. The traditional textile industry needs to improve efficiency and competitiveness by reducing costs. Defects in fabrics can reduce the selling price of manufacturers’ products by 45% to 65%, and defect detection can improve product quality to determine whether products deviate from the given specifications and improve production efficiency [1,2]. At present, the main fabric defect detection method is manual inspection. The inspection is visual and is carried out by highly trained inspectors. However, different inspectors may be affected by time consumption, subjective factors, and fatigue, and because the basis of human inspection depends on personal experience, there are no absolute criteria. Only about 70% of defects can be detected by highly trained inspectors [3,4].

The purpose of this designed turnkey automatic hardware and software integration system is to replace the current manual visual inspection process; improve the accuracy, consistency, and speed of inspection; reduce the labor cost; and improve product quality. This literature review is based on the technical bottlenecks that need to be overcome and the corresponding key technologies to be developed.

### 1.1. Noise Filtering of Woven Images

The texture features of woven fabric images are small and complex, and the noise caused during the image acquisition process causes difficulty in processing woven fabric images. There are many kinds of woven textile. Banaszczyka et al. [5] showed that using infrared imaging, the contact resistance of the fabric can be measured. Bai et al. [6] showed that vibrothermography can be used study the multiple micro cracks in the woven fabric. The goal is to find the sensitivity of the vibrothermography effect to crack density. Cho et al. [7] used an adaptive threshold for the detection of fabric defects, showing that proper noise filtering can obtain better quality images and preserve image details. Kennedy [8] applied different filters to remove the noise from signal images, showing that Gaussian filters can preserve detailed texture features and have good isotropy. Dal Moro et al. [9] applied Gaussian filters to deal with flexural and torsional vibration patterns and showed that Gaussian filters are effective in removing noise and preserving the fine features of the images. Osadebey et al. [10] used a Gaussian filter to process medical images and showed that it can remove noise from images and highlight the image edge information for subsequent image recognition.

### 1.2. Brightness Correction of Woven Image

In the process of the dynamic image acquisition of woven fabric defect images, the time of image acquisition and light source cause the difference in the color of the images, which affects the quality of the images and the extraction and recognition of features. In order to eliminate the color difference and avoid the difficulty of subsequent defect detection, the brightness correction of the woven image is the key to defect recognition.

Traditional local masking algorithms are mainly divided into two categories: masking dodging algorithm and masking proportional algorithm. Li et al. [11] proposed a masking dodging algorithm to improve the photo uneven illumination, using a low-pass filter to filter out part of the image details, proving that uneven light sources can effectively eliminate the image illumination unevenness problem. In order to increase the overall contrast enhancement, the contrast stretching method is used. However, the stretching method can only enhance the contrast to a certain extent, but it cannot completely separate the background from the defects, and it is still difficult to extract the fabric defects completely. Sun [12] mentioned that the mask dodging algorithm has its limitation. If the contrast of the original image is weak, the contrast of the image is still weak after the luminance correction, and the mask proportional algorithm is proposed to improve the background image. Zhang et al. [13] proposed a modified algorithm based on the mask dodging algorithm and the mask proportional algorithm to improve the contrast and to separate the defects from the background more clearly. However, when applied to the areas with a large contrast in grayscale values, the effect of uneven correction occurs, meaning that it cannot perform the subsequent binarization completely. Yan et al. [14] presented an improved algorithm based on the mask dodging algorithm, which used low-pass filtering and introduced a scale factor to achieve a high contrast image due to the low local contrast and uneven irradiation of the infrared optical image. The experimental results showed that the improved mask dodging algorithm can not only improve the uneven brightness of the image, but also greatly enhance the local and overall contrast of the image.

### 1.3. Extraction of Defects in the Woven Fabric

The woven fabric has a complex texture background, and it is difficult to extract the defective parts. Currently, the machine vision automatic detection system applied to woven fabric defects can be divided into three categories according to the nature of the fabric surface features: the optical spectroscopy method, the model-based methods and the statistical method [15].

#### 1.3.1. Optical Spectroscopy Method

Fabric surface texture exhibits a high degree of periodicity, allowing fabric detection using spectral features to detect defects. Texture features extracted in the frequency domain are less sensitive to noise and intensity variations than in the spatial domain. In general, the techniques commonly used for spectroscopic methods are Fourier transform and Gabor transform. Chan and Pang [16] used the Fourier analysis to detect fabric defects. The textural features extracted from the vertical and horizontal frequency components of the Fourier spectrum were used to distinguish four defect types, including double yarn, missing yarn, web, and yarn density. The application of the Fourier analysis in fabric defect detection is robust to rotation, translation and scaling, but it is unable to detect random textures [17]. When a defect occurs in fabric, its regular structure is changed so that the corresponding intensity at some specific positions of the frequency spectrum changes. However, the three-dimensional frequency spectrum is very difficult to analyze [16]. While the Gabor filter [18] is designed based on the extraction of texture features from defect-free fabric images by using a Gabor wavelet network, one of the difficulties is determining the optimal parameter setting of the Gabor filter at the same radial frequency with the high complexity of the calculation [19].

#### 1.3.2. The Model Based Approach

The model-based approach is used to construct a model based on the image, and to perform texture recognition and texture synthesis for fabric images that may have surface changes due to defects such as broken yarns and broken needles [20]. The most commonly used model-based defect detection method is the Gauss–Markov random field (GMRF) model, which measures the inter-pixel relationship by calculating the density value of each pixel on a local area [21]. Cohen et al. [22] used GMRF to simulate the texture images of non-defective fabric. However, it is susceptible to ambient light source variations and noise and does not easily identify small defects on the fabric. The model-based method is usually effective in detecting fabrics with a regular pattern texture, but the detection accuracy depends largely on the constructed model and is easily affected by environmental light source changes, which can easily lead to random changes in the actual fabric samples, resulting in a high error rate.

#### 1.3.3. Statistical Method

The statistical method is used to measure the spatial distribution of pixel values and divide the image of the inspected fabric into areas with different statistical behaviors. An important assumption in this process is that the statistics of the defect-free area are smooth, and the statistics of the area with defects are preset to have a significant difference compared to the defective area; this difference is used to determine the presence of defects, which are classified as first-order, second-order and higher order grayscale statistics [23]. The first-order grayscale statistical method of detection compares the grayscale value of each pixel in the image with the reference threshold value. If the grayscale value is greater than the threshold value, then the area has a defect, otherwise it is background. The fabric defect detection is performed directly by the signal variation of the grayscale value [24]. Hanbay et al. [25] pointed out that the detection by the first-order grayscale statistical method has the advantage of being easy to implement. Stojanovic et al. [26] developed a fabric inspection system based on the first-order grayscale statistical method by removing noise through a local average filter and then segmenting it using adaptive thresholds, followed by black vertical error, white vertical error, wrinkle, black horizontal error, and black vertical error. However, its classification accuracy is only 86.2% and it cannot identify small defects, so it is not applied to the actual production line. Cho et al. [7] mentioned that if a grayscale value exceeding the threshold value is detected in the image, the size and location of the defect in the image are stored. Such a study is designed to identify defects with a width and length over 1 mm and apply them to five representative defects. The hole and spot have high defect detection rates, but the warp float and broken pick do not, due to the image noise. Çelik et al. [27] proposed an algorithm with linear filters and morphological operations for automatic fabric defect detection running at a machine speed of 7.5 m/min. The algorithm is applied off-line and in real time to denim fabric samples for five types of defects. The defect detection performances of real-time and off-line applications are obtained as 88% and 83%, respectively. The defective images are classified with an average accuracy rate of 96.3%. However, the algorithm’s running speed cannot reach the speed of traditional machines, and the defect detection rate is too low to be practically used in the textile industry. The most commonly used method for the estimation of second-order and higher order statistics is the grayscale co-occurrence matrix. Raheja et al. [28] indicated that although the grayscale co-occurrence matrix is less than a quarter of the calculation amount of the Gabor filter, it is easily affected by the environment. Xiaowei and Xiujuan [29] presented that the computational complexity of the grayscale co-occurrence matrix is too large, making this method unsuitable for real-time systems.

### 1.4. Identification and Classification of Defect Types of Woven Fabrics

In terms of classifier, Çelik et al. [30] developed a machine vision system to achieve fabric inspection and defect classification processes. The defect detection is based on wavelet transform, double thresholding binarization, and morphological operations. The defect classification is based on a gray-level co-occurrence matrix and feed-forward neural network. The defective and defect-free regions of the fabric were shown for five commonly occurring defect types. Basu et al. [31] applied a principal component analysis to extract features from the training and test structure images, and a SVM classifier was used to perform the classification. The images were tested in the TILDA database of fabric defect standards; however, they were static. Abdellah [32] identified and classified different types of defects based on geometric features, using a genetic algorithm to search for penalty factors and kernel parameters to obtain the SVM classifier parameters under limited sample conditions. A total of 78 samples were tested for the classification of five types of defects. Al-Anazi et al. [33] constructed a model, the SVM, and a back-propagation neural network (BPNN) and generalized regression neural networks (GRN) were used in the experimental process. The experimental results showed that the classification effect of SVM is better than the others when targeting non-linear data points.

The most recent study showed similar interest in the field of fabric defect detection. Yue et al. [34] used the YOLOv4 network for the detection of defects in the fabric. They improved the detection accuracy by using data augmentation and clustering. Kahraman and Durmuşoğlu [35] proposed a capsule network, an alternative to the convolutional neural network for defect detection. The model was trained with the TILDA dataset and compared to mainstream deep learning models. Rippel et al. [36] assessed different models for fabric anomaly detection. The results showed that the techniques used in the method improve the model resistance as well as the generalization of the supervised learning technique. Xiang et al. [37] proposed an improved CenterNet model with deformable convolution for the detection of fabric defects. The study also showed the ablation technique to achieve the better performance result with a different parameter setting. These studies showed significant improvement in the field of automatic fabric quality checking; however, they require a large number of datasets in order to train the models, which is not publicly available for all kinds of defects.

In order to improve the productivity in actual woven fabric production lines, this study aims to identify and classify the eight most common defects of woven fabric to replace the current manual inspection process, in order to reduce the possibility of misjudgment and enable manufacturers to determine more quickly the defects in the process of weaving. To achieve this objective, the aforementioned technical bottlenecks must be overcome, and the corresponding key technologies must be developed.

## 2. Testing Needs of Fabric Finishing Manufacturers

**Figure 1:**
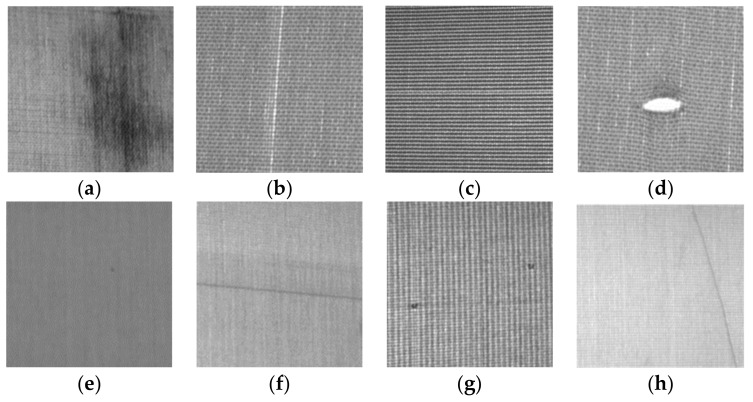
Eight mostly common occurring types of defects: (**a**) stain; (**b**) broken end; (**c**) broken weft; (**d**) hole; (**e**) nep; (**f**) double pick; (**g**) kinky weft; (**h**) float.

We visited some famous woven fabric finishing of home textile manufacturers in Taiwan. Fabric inspection is a necessary process to inspect all kinds of cotton, wool, linen, silk, chemical fiber and other large fabrics before production in the garment industry. At present, most of the textile factories use the manual fabric inspection method, relying on the experience and high concentration of the fabric inspector, who cannot conduct continuous and fast inspection due to the limitation of human eye physiology. It is not easy to recruit and train the inspectors and solve the difficulties of low efficiency, high leakage rate, unstable and inconsistent inspection results, low traceability, and management of defect information. The algorithm of the general automatic fabric inspection system remains confidential, and the price is too high for the general textile enterprises to afford. In addition, each textile manufacturer’s garment product has its own characteristics, and the origin of the fabric and the type and quantity of defects it contains are different, so the design of the fabric inspection machine needs to meet the specific necessities of each production inspection and economic efficiency. In this study, eight types of defects (stain, broken end, broken weft, hole, nep, double pick, kinky weft, and float), which were obtained from the home textile manufacturer’s woven fabric finishing survey, were designed, as shown in Figure 1.

### 2.1. Fabric Defect Definition

The defect definitions of the appearance of fabrics are as follows:

(1) Stain: Those with oil stains on the cloth.
(2) Broken end: One or more warp yarns of the fabric are broken, causing the distance between the left and right adjacent yarns to increase.
(3) Broken weft: The weft in the fabric is broken, but the two ends of the break are very close, that is, the length of the break is small.
(4) Hole: The warp and weft yarns of the fabric are broken, forming holes of different sizes. Such defects are prone to occur in fabrics with dense warp and weft.
(5) Nep: Thick sized balls are tightly knotted on the cloth.
(6) Double pick: Two weft yarns are woven into the same weave mouth, and there are also three or more weft yarns.
(7) Kinky weft: The weft of the fabric has a small section that is crimped and twisted together and woven into the fabric.
(8) Float: The warp or weft yarns are not woven in accordance with the prescribed organization, but float on the surface of the cloth.

### 2.2. Detection of Cloth Species

**Figure 2:**
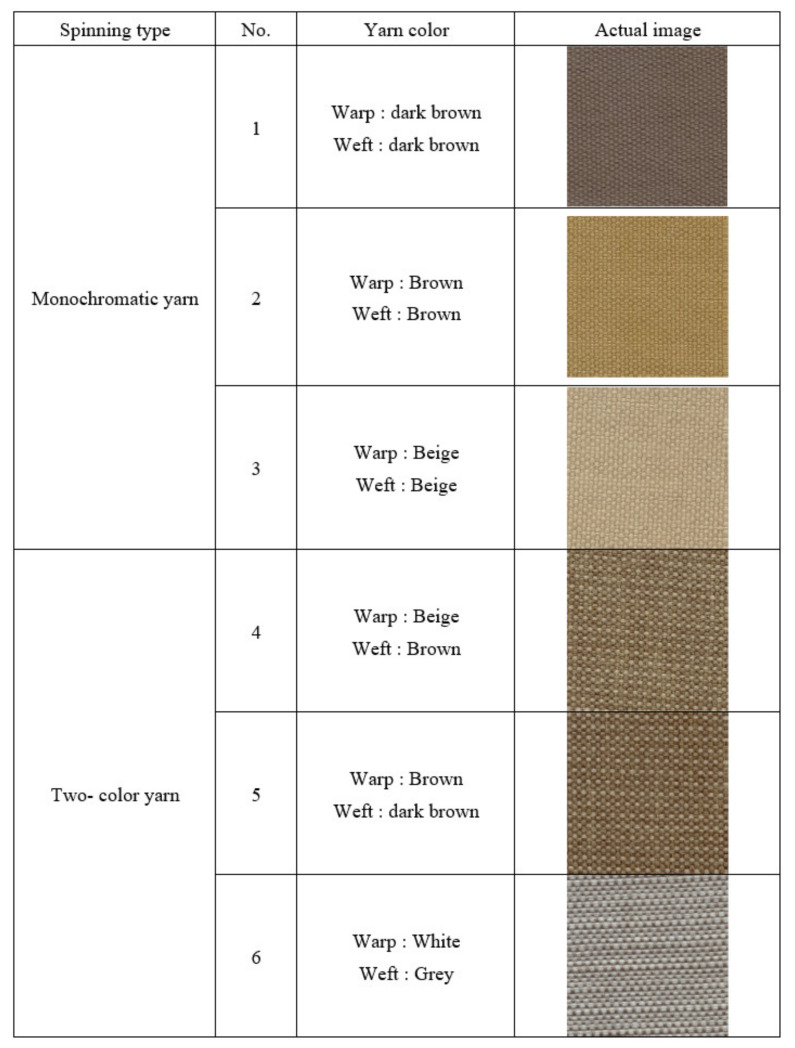
Detection of cloth species.

Due to the diversity of the weave and color of woven fabrics, six samples of different colors of yarn and weave were selected for testing, as shown in Figure 2. Therefore, the calculation developed in this study is the robustness to color and weave.

## 3. Methodology

This research concerns the development of fabric defect detection and classification. The captured images undergo image preprocessing to filter out noise, correct brightness, and be enhanced. Then, the images are binarized to segment the background and defects. The defects are filtered out through a small area using the morphological method. The position of the defect in the image and the feature of the defect are confirmed, and then the extracted feature is input into the classifier for classification, in order to achieve defect identification and classification.

### 3.1. Brightness Correction

In the process of image capture, due to external environmental interference factors, the refraction and reflection of light occurs, resulting in a ring halo in the image, which is captured by the complementary metal oxide semiconductor (CMOS) industrial camera. The phenomenon of uneven illumination occurs when the image has different degrees of difference in brightness. Halo affects the captured images to varying degrees and has a great impact on the subsequent defect processing. Therefore, it is necessary to deal with the halo and eliminate the uneven illumination of the fabric image to make it high quality.

According to the principle of uniform light [11], the image with uneven illumination is expressed as Equation (1):

(1) $$I^{\prime }(x,y)=I(x,y)+B(x,y)$$

where $I^{\prime }(x,y)$ represents images with uneven lighting, f(x,y) represents an image that receives uniform light under ideal conditions, and B(x,y) represents the background image. From Equation (1), it can be known that the image with uneven illumination can be regarded as the result of superimposing a background image on an image with uniform illumination. The uneven halo phenomenon in the captured image is caused by the unevenness of the background image. Therefore, if the background image of the image can be generated, the uneven halo can be removed from the original image to obtain a uniformly illuminated image.

### 3.2. Improved Algorithm of Mask Dodging

This study applied an improved algorithm of mask dodging [14] to successfully extract the segmentation binarization and avoid image distortion. The purpose was to improve the contrast and separate the defects from the background more completely, so that the subsequent binarization could be performed. When converting, it can completely extract part of the defective image.

**Figure 3:**
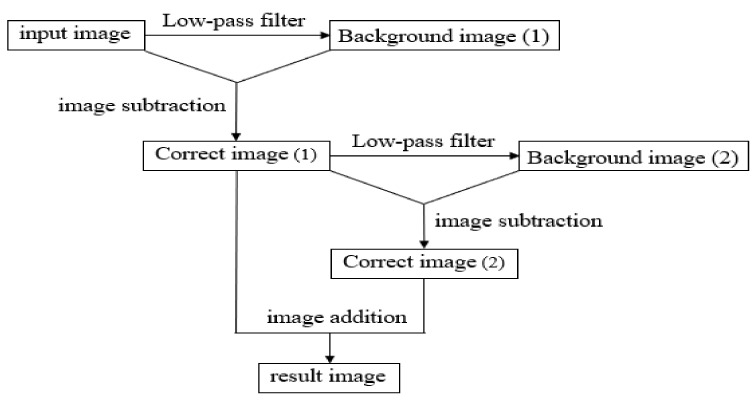
Flowchart of the improved algorithm of mask dodging.

The flowchart of the improved algorithm of mask dodging is shown in Figure 3. The first background image (1) *g*_1_*(x,y)* is generated by passing the input original image *f*(*x,y*) through a low-pass filter, and then subtracting the input original image *f(x,y)* from the background image (1) *g*_1_*(x,y)* to obtain the corrected image (1) *z*_1_*(x,y)* after applying the mask dodging method, which solves the problem of the uneven light source. In order to improve the issue of low contrast, the obtained image is then blurred through a low-pass filter to generate a background image (2) *g*_2_*(x,y),* and by subtracting the corrected image (1) *z*_1_*(x,y)* and the background image (2) *g*_2_*(x,y)*, the corrected image (2) *z*_2_*(x,y)* can be obtained; its image mainly contains high frequency, that is, to correct the details of the image (1) *z*_1_*(x,y)*. Finally, the correct the image (2) *z*_2_*(x,y)* is obtained by multiplying the corrected image (1) *z*_1_*(x,y)* to increase the contrast of the image. After the above steps, the resulting image *I(x,y)* can be corrected well, and its algorithm can be expressed by Equation (2).

(2) $$\begin{array}{l} I(x,y)=(f(x,y)-g_{1}(x,y))+\lambda \times (z_{1}(x,y)-g_{1}(x,y))\\ \;\;\;\;\;\;\;\;\;\;\;=z_{1}(x,y)+\lambda \times (z_{1}(x,y)-g_{2}(x,y))\\ \;\;\;\;\;\;\;\;\;\;\;=z_{1}(x,y)+\lambda \times z_{2}(x,y) \end{array}$$

The image captured in this study is a grayscale image; the improved algorithm of mask dodging is used to improve the uneven brightness in the image.

### 3.3. Average Correction

The average correction is to calculate the average value of the gray-level histogram of each image and divide the gray-level value 128 by the average value of each image as the basis for translation, as shown in Equations (3) and (4). Each image after translation can segment the original image on the same benchmark in the subsequent binarization. The average grayscale value of the woven fabrics discussed in this study is between 120 and 215, and the same set of algorithms can be used for defect detection and classification.

(3) $$mean=\frac{1}{M\times N}\sum_{i=1}^{M}\sum_{j=1}^{N}I(i,j)$$

(4) $$I_{new}(i,j)=\frac{128}{mean}\times I(i,j)$$

### 3.4. Image Features

In this study, the following features are determined according to the four characteristics of independence, distinction, reliability, small defect size and the characteristics of the actual defects after segmentation, including area, average grayscale value, defect directionality and aspect ratio. The image features are used in the SVM classifier to detect the defect in the image.

#### 3.4.1. Area

The calculation of the area is the result obtained by adding up the pixels that mark the same area after the image is subjected to the connected labeling method. The perimeter is the sum of the pixel values of the peripheral contour after the pixels marking the same region are circled by the contour.

#### 3.4.2. Average Grayscale Value

To obtain the average gray-level value is to mark the image by the connected component labeling, record the coordinate position of the marked same object, correspond to the original image, add up the pixel values of the corresponding gray contact points, and finally divide by the number of pixels. The average grayscale value can be obtained, and the calculation formula of the average grayscale is shown in Equation (5):

(5) $$\bar{f}=\frac{1}{S_{num}}\sum_{f\subset S}f(x,y)$$

where $\bar{f}$ is the grayscale average value of the obtained connected component labeling, *S* is the set of connected component labeling, and $S_{num}$ is the number of sets of connected component labeling.

#### 3.4.3. Aspect Ratio

The aspect ratio can describe the ratio of the outer contour of the image object. It is used to mark the image within the smallest rectangle area. The ratio of the long axis and the short axis of the image object can be obtained as:

(6) Aspect ratio=L/W

where *L* is the length of the frame selection area, and *W* is the width of the frame selection area.

#### 3.4.4. Defect Directionality

**Figure 4:**
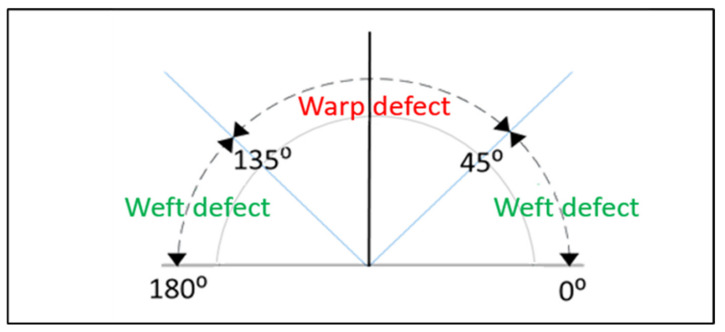
Defect directionality: range of angles of warp and weft defects.

Since the woven fabrics are woven by warp and weft yarns, the defective parts of the fabric will also have the characteristic of directionality, as shown in Figure 4. The defects are classified by this feature for warp and weft defects. In Figure 4, it can be seen that if the defects direction is within 45° to 135°, then it is considered as a warp defect; otherwise, it is a weft defect.

#### 3.4.5. SVM

SVM is a binary linear classifier. It has an excellent learning ability in solving small samples and multi-dimensional data [38]. When training data or classifying, its execution speed is faster than other classifiers [39]. Therefore, SVM is used in this study as the classifier to identify the defect types.

**Figure 5:**
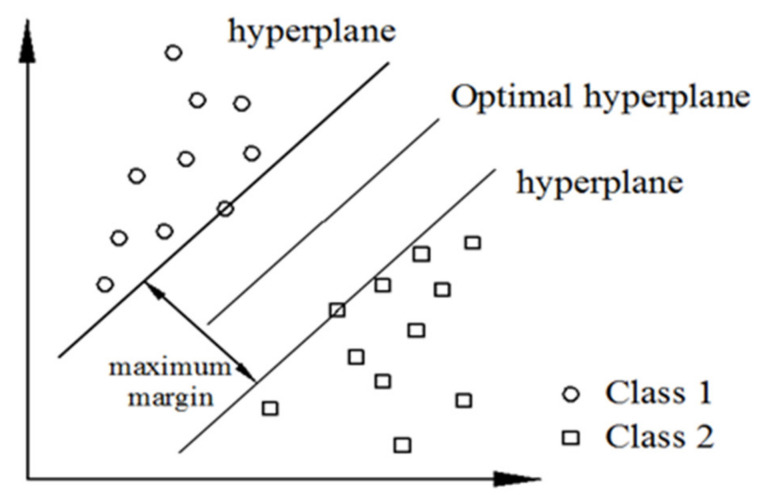
Schematic diagram of SVM.

The SVM classifier finds a hyper-plane in the space, so that two types of data can be distinguished by the hyper-plane. The hyper-plane and the point with the closest distance between the two categories are the margin. The SVM is to find a zone with the largest boundary in the sample groups of two different categories and separate the two categories of data with the optimal hyper-plane. Then, if there are unknown data to determine the category, it is determined according to the position of the data on the hyper-plane, as shown in Figure 5.

## 4. Actual Machine Design and Verification

Since the conversion of the captured image into digital information for processing involves a huge amount of information, and considering the real-time nature of the online system, the processing speed and memory capacity of the computer must be taken into consideration, and the operating system must also be universal and compatible to facilitate maintenance and updates.

### 4.1. Image Capture System and Computer Hardware

Based on the actual hardware requirements, the woven fabric defect detection system and the algorithm of defect identification will be developed to display the experimental results. The image capture hardware used are as follows:

(1) CPU: Intel core (TM) i7-6700 CPU 3.40GHz.
(2) 16.0GB random access memory.
(3) The Visual C++, Common language runtime, and open-source computer vision library are used as software development tools.
(4) Optical magnification lens and LED module.
(5) Industrial camera: Basler acA4600-10uc, CMOS area scan camera, 14 MP resolution, USB 3.0 camera interface, rolling shutter.

For the cloth rolling machine, in the light source selection, the light source of the woven fabric detection system needs to be used for a long time, so LED lights are selected as light sources. Compared with traditional halogen lamps and incandescent lamps, LED lights have the characteristics of long life and high luminous efficiency, and the light source will not flicker.

In the lighting, if simply using the ambient light source, the image features obtained are not obvious, so it is necessary to use a set of light sources to enhance the image features. In this study, the front light source and the backlight source are used at the same time to enhance the characteristics of the image and reduce the interference of external light sources. The reason for this is that the fabric defects can be divided into bright defects and dark defects, and the front light source can highlight the changes in the structure of the fabric defects. The shadows or dark defects (nep, kinky weft, float, stain) emerge, and the backlight highlights the bright defects (broken end, broken weft, hole). Therefore, this study also used front light source and backlight source for detection.

### 4.2. Operating System

This research used Microsoft Windows 7 64 bit as the operating system to develop a user-friendly interface. The above-mentioned hardware equipment is used as the operating environment for the program development to speed up the operation time of the image processing software.

### 4.3. Program Development Software

Microsoft Visual studio 2019 was launched by Microsoft as a development tool. The Visual studio integrated development environment provides a good C++ programming tool, packaging various functions into control items. Developers only need to drag the control items to the design window; the studio quickly develops related applications, reduces the need to write programs to describe the appearance and configuration of the input and output interfaces, and greatly reduces the development time of programmers.

Using OpenCV as the program development platform—because it contains many functions related to computer image conversion, image processing, and mathematical operation processing—can greatly shorten the development time, and the friendly development environment can help users to debug quickly. OpenCV also has complete file format support. There are also general algorithms for image processing and machine vision, which can be quickly executed and practically applied.

**Figure 6:**
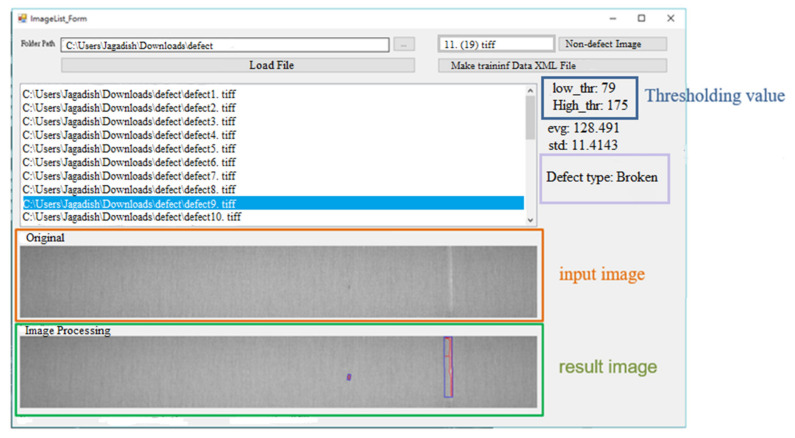
Graphical user interface for the developed system.

Through the hardware equipment and software development platform, this research develops a fabric defect detection system and designs a user-friendly human–machine interface, as shown in Figure 6.

### 4.4. Experimental Machine Architecture

**Figure 7:**
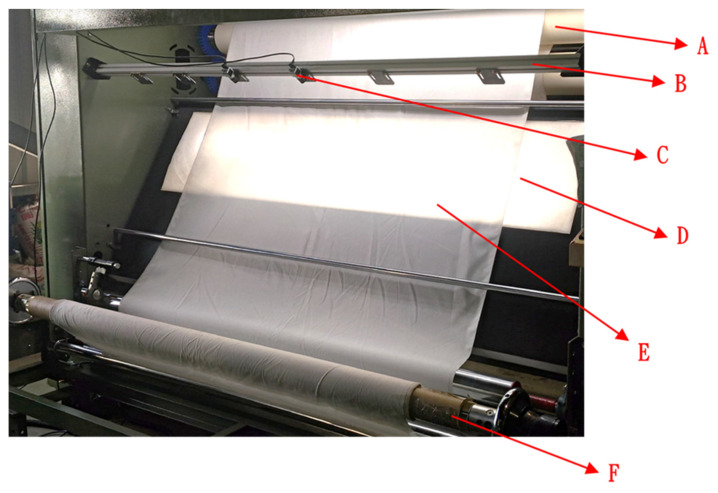
Actual structure of the experimental machine.

**Figure 8:**
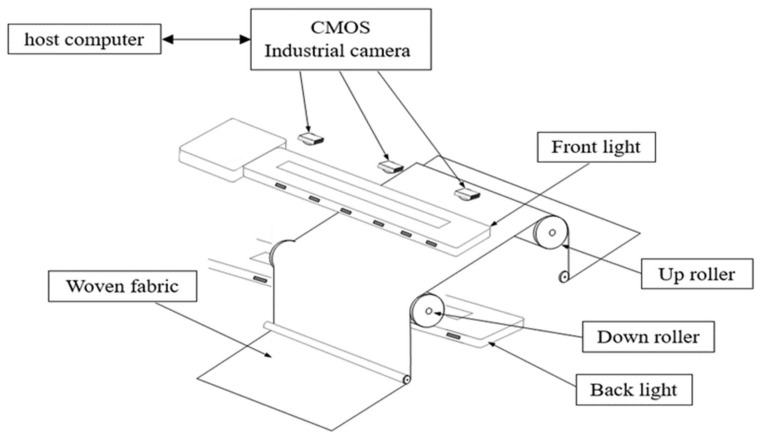
Schematic diagram of the experimental machine.

The structure of the experimental machine in this study is shown in Figure 7, where A is the upper reel, B is the front light, C is the CMOS camera, D is backlight, E is the woven fabric, and F is the lower reel. Figure 8 is the woven fabric defect detection system developed in this study, which detects eight common types of defects. The image acquisition process of the woven fabric defect detection system is as follows:

First, the woven fabric is placed between the upper reel and the lower reel; the CMOS industrial machine is located above the woven fabric. In the light source, the front light source is placed above the woven fabric, and the backlight is placed under the woven fabric. A computer program is used to control the machine. The woven fabric is moved from the upper reel to the downward reel and sampled with a CMOS industrial camera; then, the captured image is determined by the developed image processing algorithm to determine whether there is any defect inside the detected woven fabric. The classifier is used to identify the defect type.

### 4.5. Defect Detection Process

**Figure 9:**
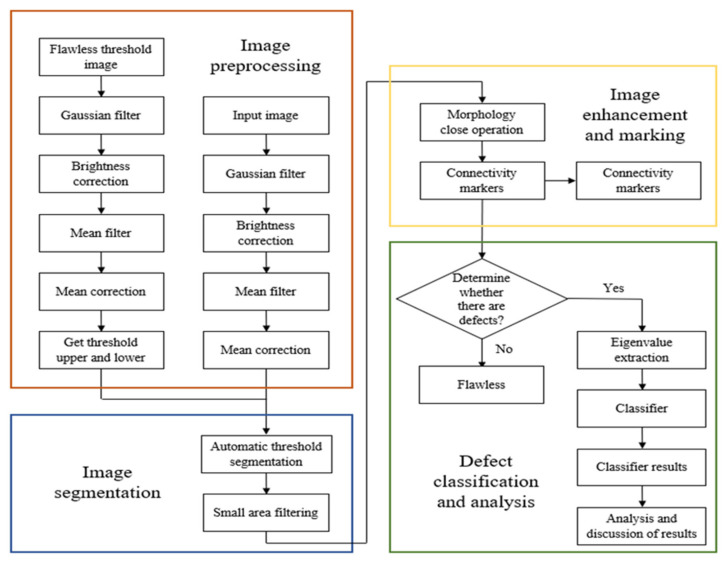
Image processing flow chart.

In this study, image processing is used in the detection of woven fabric defects, and eight kinds of defects including stain, broken end, broken weft, hole, nep, double pick, kinky weft, and float are detected. The image processing is divided into four parts: image preprocessing, image segmentation, image enhancement and marking, and detect classification and analysis. The detailed image processing flow is shown in Figure 9. The first step of the defect identification system for woven fabrics proposed in this study is to use a CMOS industrial camera to capture images and select one of the flawless images to produce a brightness-corrected image. The second step is to input a flawless image to filter out the noise through a Gaussian filter. As the image contains uneven brightness, we use the brightness correction image created in the previous step to perform the algorithm for brightness correction, and then use the mean filter to filter out the background texture. Finally, through the average correction method, the image histogram is shifted, and the average gray level of the image is adjusted to the gray-level value of 128. Then, the mean and standard deviation of the flawless image is calculated to establish the binarization segmentation threshold, and the image preprocessing part is completed. The third step is to perform segmentation by the binarization threshold established in the second step to separate the foreground (blemish) part from the background (non-blemish) part. Then, the incomplete segmentation is modified through the morphological closing operation, so that the segmentation result can be more complete. The fourth step is to filter out noise points that are too small through small area filtering and calculate the number of defects through the connected component labeling, then select each defect position with a red line circle. The fifth step is to judge whether there is a defect through the statistics of the number of defects and extract the eigenvalues for each defect. The sixth step is to input the eigenvalues into the classifier for defect identification to determine eight types of defects.

#### 4.5.1. Capturing Sample Images

**Figure 10:**
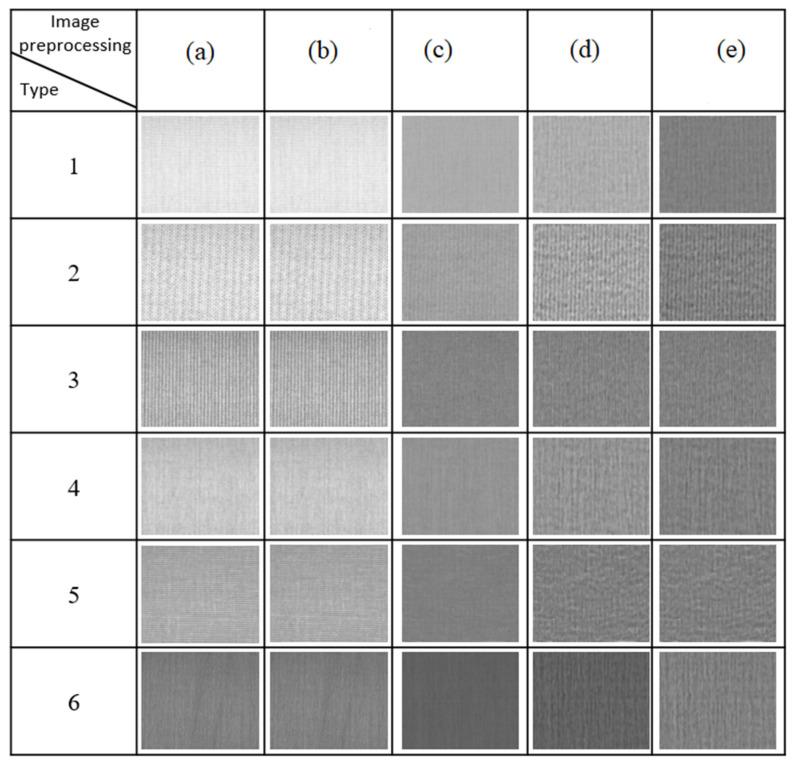
The image preprocessing demonstration: (**a**) Grayscale image; (**b**) Filtering image; (**c**) Brightness correction; (**d**) Contrast upgrade; (**e**) Average correction.

In this study, two light sources, the front light source and the backlight source, were used for lighting. The front light source can highlight the shadows or dark defects produced by the structural changes of fabric defects (nep, kinky weft, float, stains); the backlight is mainly used to highlight the bright defects (broken end, broken weft, hole). The six woven fabrics were taken and captured separately as grayscale images. The size of each image was 4600 × 600 pixels. The grayscale images for the six types of fabrics are shown in Figure 10a.

#### 4.5.2. Filter Out Image Noise

During image capture, noise is generated due to factors such as environment or signal noise. In this study, a Gaussian filter is used to filter out the noise in the image to avoid misjudgment. To reduce the subsequent segmentation errors, the resulting image after filtering out noise is shown in Figure 10b.

#### 4.5.3. Brightness Correction

The woven fabrics tested in this study are subject to the refraction and reflection of light due to external environmental disturbances, resulting in a ring halo in the image during the image capture process, resulting in varying degrees of brightness in the captured image, which has a great impact on the subsequent use of image processing to detect defects in the halo area. In order to avoid the problem of indistinguishable defects caused by an uneven halo, this section uses an improved mask difference algorithm to eliminate and reduce the halo phenomenon. The modified mask difference algorithm is shown in Equation (7). Figure 10c shows images that were taken with both the front light source and the backlight source after Gaussian blurring.

(7) $$I(x,y)=(f(x,y)-g_{1}(x,y))+\lambda \times (z_{1}(x,y)-g_{2}(x,y))$$

where *f* (*x*,*y*) is the original image grayscale value, I(x,y) is the image pixels corrected by the modified mask difference algorithm, *g* (*x*,*y*) is the background image pixels generated by the low-pass filter, *z* (*x*,*y*) is the corrected image pixel point after correction, and *λ* is the contrast ratio, which is usually set in the range of 0 to 5. In this study, *λ* was set to 2.5. The image contrast upgrade is shown in Figure 10d.

#### 4.5.4. Image Average Correction

Since the difference in the color of the woven fabrics also causes errors in the judgment of defects, this study performs an average correction for each image. Through Equations (3) and (4), the algorithm is used for defect detection and defect classification, so the fabric defect detection system proposed in this study has good adaptability to the color of the fabric. The average correction results are shown in Figure 10e.

#### 4.5.5. Image Segmentation

**Figure 11:**
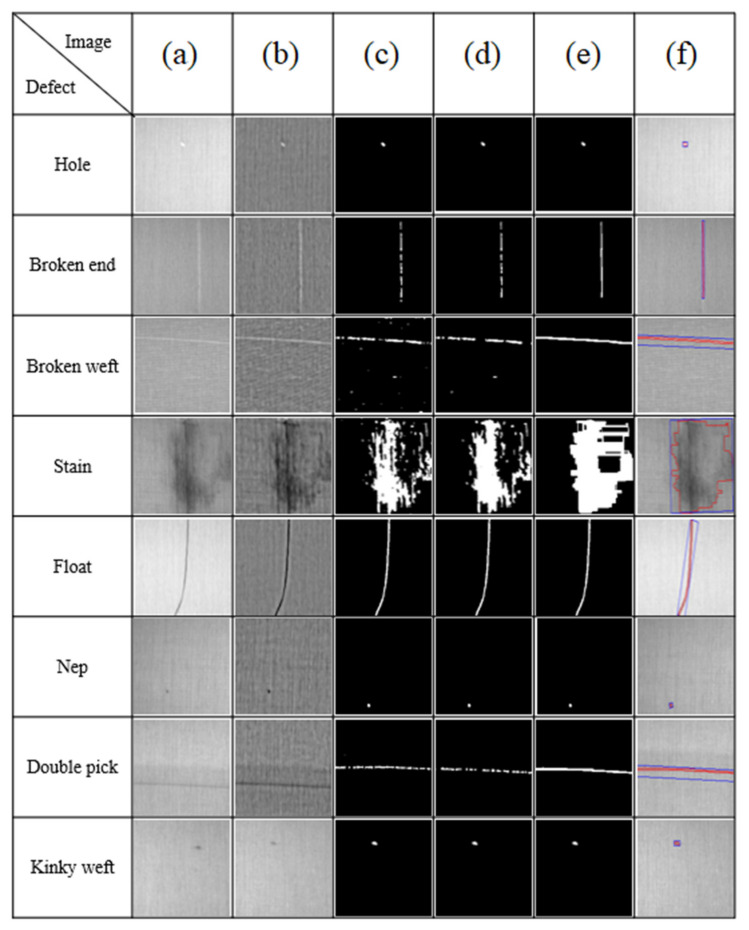
The image segmentation demonstration: (**a**) Original image; (**b**) Image preprocessing; (**c**) Binarization split image; (**d**) Smaller area filter out; (**e**) Morphology closed operation; (**f**) contour selection.

We used the average and standard deviation of six woven fabrics to generate subsequent images after image preprocessing. During adaptive binarization, the upper and lower thresholds are required to segment defects and backgrounds. Then, the obtained threshold is used directly for threshold segmentation, followed by the closed operation method in morphology to fill in the incomplete part of the binarized segmentation, so that eight types of common defects in woven fabrics can be made more accurate during image segmentation. The complete segmentation result has a great influence on the subsequent feature value extraction and classification. The detailed process of this research through adaptive binarization and morphological closing operation is as follows:

First, when the CMOS industrial camera captures the image as shown in Figure 11a, it undergoes the pre-processing steps, as shown in Figure 11b, which can eliminate the problem of halo phenomenon and improve the overall image contrast, so that the defects and the background can have better grayscale values. The large difference helps the image to return better segmentation results in subsequent segmentation.

Second, adaptive binarization is used to segment the imperfections and background. As the histogram distribution of the image after brightness correction will be close to the normal distribution, it can be seen from the characteristics of the normal distribution that about 99.7% of the area in the image will appear within three standard deviations of the mean, and the defects are abnormal points in the image. Thus, when using the threshold value for segmentation in this study, three standard deviations around the mean are used as the upper and lower limits of segmentation, as shown in Equation (8). When the grayscale value in the image is not within the control, the grayscale pixel is 255. On the contrary, it is 0, and the results of the image after adaptive binarization segmentation are shown in Figure 11c.

(8) $$g(x,y)=\left\{\begin{array}{c} \begin{array}{lll} 255 & if & \mu -3\sigma \leq u\leq \mu +3\sigma \end{array}\\ \begin{array}{ll} \;\;\;\;\;\;\;\;\;\;\;\;\;\;0 & otherwise \end{array} \end{array}\right.$$

where μ is the image gray-level average, σ is the image standard deviation.

Filtering out a small area can separate the background and defects after binarization, but the image after binarization will have a lot of noise. This noise is caused by the excessive pores of the yarn or the influence of noise, so the action of the defect area is too small to avoid subsequent misjudgment or over-grabbing. Therefore, this study sets that if the area of the defect is less than five pixels, that is, when the area of the defect is less than 0.5 mm the block will be filtered out. The required results are shown in Figure 11d.

#### 4.5.6. Image Enhancement and Connectivity Marking

This section introduces the confirmation and circle selection of the defect range. Then, in order to repair the damaged part of the binarized image, the incomplete circled part is filled through the morphological closing operation, and the defect is marked by the Unicom marking method. As the basis for the number of defects, finally, the position of the defect is completely circled by the method of contour circle selection, and the characteristic value of the defect is calculated for the connected marked area. Then, the characteristic value is imported into the subsequent classifier for classification.

The first step is to use morphological operations. Since there may be discontinuous line segments after the threshold segmentation, it is necessary to use closing and opening to repair the discontinuity or incomplete segmentation. In this study, the morphological closing operation is used, as shown in Figure 11e.

In the second step, through the method of eight connections, the connected marking method is used to calculate the number of defects, and the number of defects is used as a basis for confirming whether the current image has defects. Then, through the method of edge detection and contour circle selection, the position of the defect is marked; the marking results are shown in Figure 11f.

#### 4.5.7. Analysis of Segmentation Results

In this study, the detection success rate, detection rate, and false alarm rate [40], as shown in Equations (9)–(11), are used as the performance evaluation for the segmentation results.

(9) $$Detection\;Success\;Rate=\frac{\mathrm{TP}+\mathrm{TN}}{\mathrm{TP}+\mathrm{TN}+\mathrm{FP}+\mathrm{FN}}$$

(10) $$Detection\;Rate=\frac{\mathrm{TP}}{\mathrm{TP}+\mathrm{FN}}$$

(11) $$False\;Alarm\;Rate=\frac{\mathrm{FP}}{\mathrm{TN}+\mathrm{FP}}$$

The non-defective images are defined as (Positives), defective images as (Negatives), detection results as normal (True), and detection results as defective (False), where TP is true positives, FN is false negatives, and TN is true negatives (TN).

In this study, a total of 2246 images were extracted from the six samples, of which there were 1810 images of non-defective images and 436 images of defective images as the sample number. The detection success rate is 96.44%, the detection rate is 96.35%, and the misjudgment rate is 3.21%. The results show that the performed image preprocessing, image enhancement and adaptive binarization can effectively detect defects. The algorithm is also designed to work in a relatively low light condition. However, if the light intensity is very low, the algorithm cannot work properly. Apart from this, the algorithm can work properly to identify a defective or non-defective sample even though the sample may have noise because of the image processing technology applied to it. A relatively small defect can also be identified as kinky weft, which is shown in Figure 11. Therefore, the algorithm can be implemented in the fabric quality inspection system for better quality product output.

#### 4.5.8. Defect Feature Analysis

**Figure 12:**
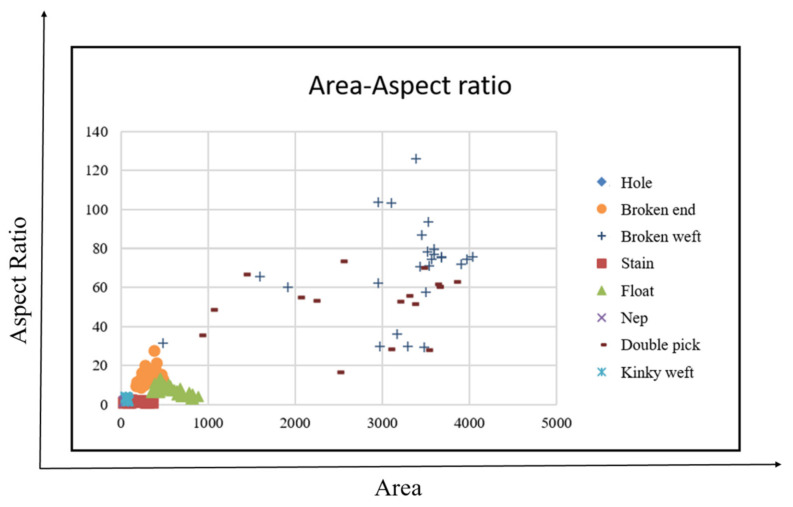
Area versus aspect ratio feature distribution.

**Figure 13:**
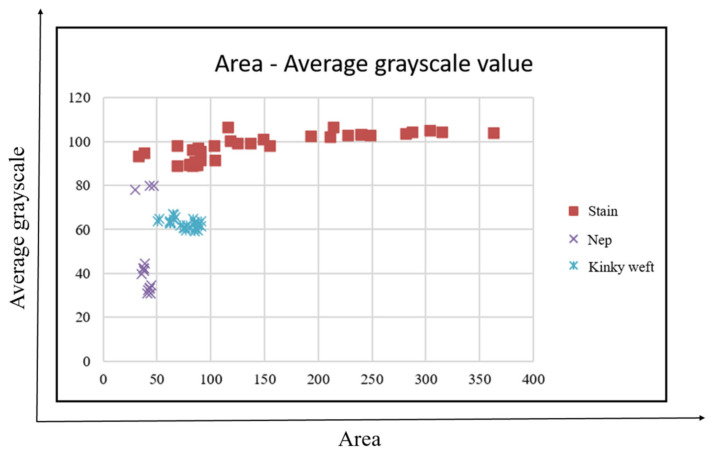
Area vs. average grayscale value feature distribution.

**Figure 14:**
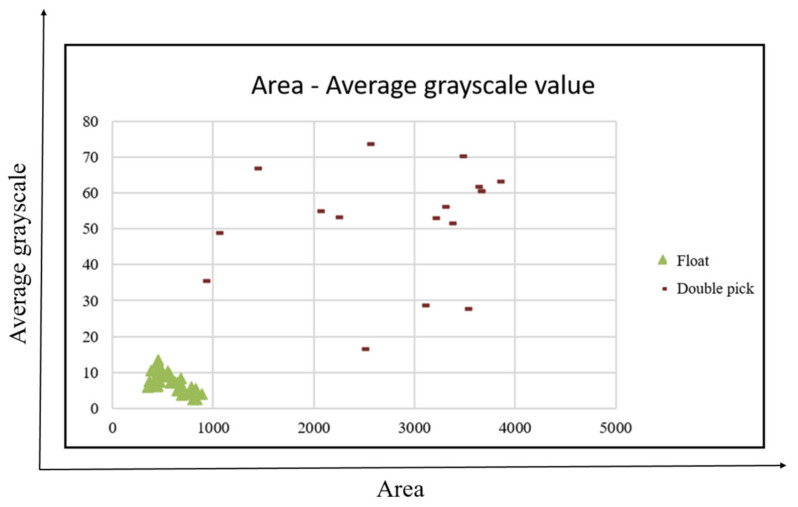
Area vs. average grayscale value feature distribution.

This study uses the definition of defects to select four features, namely, area, aspect ratio, average grayscale value, and defect directionality for analysis. A total of 230 defects are used to map the distribution of defect characteristics, that is, 21 holes, 19 broken ends, 25 broken wefts, 27 stains, 42 floats, 38 neps, 18 double picks and 40 kinky wefts, thereby obtaining the best classification results, as shown in Figure 12, Figure 13 and Figure 14.

The area of the x-axis in Figure 12 corresponds to the aspect ratio of the y-axis. Although the defects cannot be completely separated, they can be divided into two types: small area and small square defects (holes, stains, neps, kinky weft), and long defects with a large area and large aspect ratio (broken weft, float, double pick), which can assist the classifier in making a preliminary judgment. From the area of the x-axis in Figure 13 corresponding to the average grayscale value of the y-axis, the three types of stains, neps, and kinky weft can be distinguished. The area of the x-axis in Figure 14 corresponds to the average grayscale value of the y-axis, which can distinguish double pick with a larger area from float. The directional properties are used to distinguish two types of defects: broken end and broken weft. Based on the above observations, this study uses four features of area, aspect ratio, average grayscale value and defect directionality as the input of the classifier.

#### 4.5.9. Defect Classification

**Figure 15:**
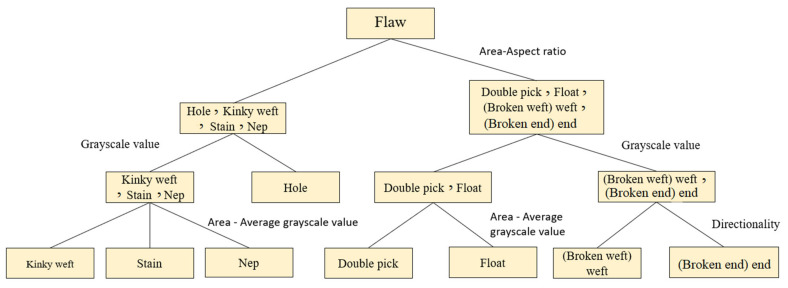
Defect classification model.

In this study, SVM is selected as the defect identification classifier in this study. The 230 training defect samples of the classifier are: 21 holes, 19 broken warps, 25 broken wefts, 27 stains, 42 floats, 38 neps, 18 double picks, and 40 kinky wefts. The 206 test samples are: 21 holes, 16 warps, 18 wefts, 21 stains, 42 floats, 38 neps, 11 double picks and 39 kinky wefts. First, the training samples are used for training; then, the model of the SVM is constructed; finally, the test samples are classified using the pre-established model, as shown in Figure 15. The classification results are shown in Table 1.

Table 1 shows that the identification rates of the SVM in this study are 100% for holes, 100% for broken ends, 100% for broken wefts, 93.02% for stains, 97.62% for floats, 92.10% for neps, 100% for double picks, and 94.87% for kinky wefts. The overall recognition rate is 96.60%, which represents that the selected classifier can effectively classify defects.

## 5. Discussion

### 5.1. Comparison of Traditional Woven Fabric Defect Detection

The traditional woven fabric inspection method is carried out visually by the human eye. However, this type of inspection has many shortcomings: the human eye grows tired easily; the detection rate of small defects is too low; and the inspection standards are inconsistent. In this study, an automated woven fabric defect detection system is proposed to improve the above-mentioned problem of insufficient human eye detection through machine vision. Table 2 presents the comparison between manual detection and the automatic detection method proposed in this study.

### 5.2. Comparison Studies

Table 3 shows the comparison study with other related publications. The study shows that our method can more effectively identify the defects than other studies. Our study achieves a 96.60% defect detection rate; Basu et al. [31] achieved 96.36%, whereas other studies [30,32] achieved less than 95%. Our study also has a greater number of defect types than other studies. Our study achieves a relatively good detection rate due to the implementation of the adaptive method in image processing technology, as well as the segmentation method and image morphology, which enhance the method and enable it to perform better than others.

### 5.3. Machine Speed Evaluation

**Figure 16:**
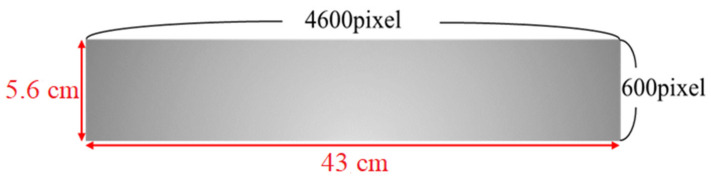
The relationship between the pixels and the actual size of the captured image.

In this study, the grayscale image of the woven fabric captured by the CMOS industrial camera has an image size of 4600 × 600 pixels, which is relative to 43 × 5.6 cm, i.e., the real image size, as shown in Figure 16.

This study uses 500 images to test the calculation time of the algorithm for each image. The test results show that the average processing time per image is about 0.125 s, which means that the algorithm designed in this study can process eight images per second. The width of each image is calculated as 5.6 cm, so the algorithm proposed in this study can be calculated at 26.88 m/min, and its calculation time can be applied to the operating speed of the traditional machine (20 m/min).

In terms of detecting the minimum defect specification, it can be known from the above information that the relative actual image size of each pixel is 1 pixel equal to 0.0934 mm. After the image is set to be connected in this study, the experimental results show that if the image pixel size is greater than 5, the actual size is 0.467 mm, and the circled block is judged as a defect, which can filter out the noise in the image and accurately identify the small defects. Therefore, this study sets the minimum detection defect size as 0.5 mm.

## 6. Conclusions

This study proposes a set of optical inspections of woven fabric defects, including a self-designed and developed machine, as well as a digital image processing process, in order to detect eight of the most common defects in the manufacturing process and ensure the stability of the product quality. The conclusions are as follows:

(1) The experimental machine that is designed and developed in this research includes a complementary metal oxide semiconductor (CMOS) industrial camera, a light source structure and a traditional winding machine to construct a complete set of optical inspection experimental machines. The cloth winding machine runs at a speed of 20 m/min, and the size of the captured images is 4600 × 600 pixels.
(2) In this study aiming at the halo phenomenon caused by the refraction and reflection of light due to external environmental interference, the improved mask dodging algorithm is used to eliminate the uneven brightness caused by the halo phenomenon. The experimental results show that the standard deviation and uniformity of the original image are 12.072 and 47.78%, respectively, and after correction by the improved mask dodging algorithm, these become 2.891 and 73.28%.
(3) In this study, the adaptive binarization method is used for segmentation, so that the developed system can still completely segment the flaws and backgrounds under different types of cloth seeds. Image repair and enhancement are employed so that the follow-up defect identification and defect classification have good results.
(4) A total of 2246 images were extracted from six woven fabric samples, including 1810 images of defect-free images and 436 images of defective images. The detection success rate is 96.44%, the detection rate is 96.35%, and the misjudgment rate is 3.21%.
(5) This study selects area, aspect ratio, average gray value and defect directionality as the inputs of the SVM classifier. The experimental results show that the overall recognition rate reaches 96.60%.

Future studies can focus on improving the accuracy, recall, precision and fast processing of the defect inspection systems. New methods can be applied to the existing method to improve the detection model. Future scope may focus on deep learning or transformers in small datasets with the aim of improving those models for better classification and identification.

## Figures and Tables

**Table 1:** Identification results of support vector machines.

	Sample	Hole (21)	Broken End (16)	Broken Weft (18)	Stain (21)	Float (42)	Nep (38)	Double Pick (11)	Kinky Weft (39)
Results									
Hole		21	0	0	0	0	0	0	0
Broken end		0	16	0	0	0	0	0	0
Broken weft		0	0	18	0	0	0	0	0
Stain		0	0	0	20	0	0	0	0
Float		0	0	0	0	41	0	0	0
Nep		0	0	0	0	0	35	0	2
Double pick		0	0	0	0	1	0	11	0
Kinky weft		0	0	0	1	0	3	0	37
Recognition rate		100%	100%	100%	95.23%	97.62%	92.11%	100%	94.87%

**Table 2:** Comparison between the traditional detection and the automatic detection proposed in this study.

	Traditional Detection Method (Human Eye Detection)	Automated Inspection System
Detection process	1. The rolling machine is running 2. Find flaws 3. Stop the winder 4. Make a mark 5. Determination of defect types 6. Roller running	1. The rolling machine is running 2. The algorithm judges whether it is a defect 3. Find flaws 4. Record the defect location 5. Identify the type of defect
Defect overall classification rate	75%	96.60%
Speed	2 s/image	0.125 s/image

**Table 3:** Comparison with our related study.

Study	Method	Defect Detection Rate
Çelik et al. [30]	Wavelet transform, Morphological operation	93.6%
Basu et al. [31]	Computer vision, PCA method	96.36%
Abdellah et al. [32]	Genetic algorithm	94.84%
Our study	Image processing, SVM	96.60%
